# Detection of novel drug-adverse drug reaction signals in rheumatoid arthritis and ankylosing spondylitis: analysis of Korean real-world biologics registry data

**DOI:** 10.1038/s41598-024-52822-w

**Published:** 2024-02-01

**Authors:** M. Kwon, C. I. Joung, H. Shin, C. C. Lee, Y. S. Song, Y. J. Lee, S. Kang, J. Y. Kim, S. Lee

**Affiliations:** 1 Department of Internal Medicine, School of Medicine, Konyang University, Daejeon, South Korea; 2 Konyang University Myunggok Medical Research Institute, Daejeon, South Korea; 3 Department of Biomedical Informatics, School of Medicine, Konyang University, Daejeon, South Korea; 4 Healthcare Data Science Centre, Konyang University Hospital, Daejeon, South Korea; 5 Department of Pathology, School of Medicine, Konyang University, Daejeon, South Korea; 6 Department of Rehabilitation Medicine, School of Medicine, Konyang University, Daejeon, South Korea; 7 Department of Otorhinolaryngology-Head and Neck Surgery, School of Medicine, Konyang University, Daejeon, South Korea; 8 Department of Computer Engineering, Gachon University, (13120) 1342 Seongnamdaero, Sujeong-gu, Seongnam-si, Gyeonggi-do South Korea

**Keywords:** Rheumatology, Health care

## Abstract

This study aimed to detect signals of adverse drug reactions (ADRs) associated with biological disease-modifying antirheumatic drugs (DMARDs) and targeted therapies in rheumatoid arthritis (RA) and ankylosing spondylitis (AS) patients. Utilizing the KOrean College of Rheumatology BIOlogics & Targeted Therapy Registry (KOBIO) data, we calculated relative risks, excluded previously reported drug-ADR pairs, and externally validated remaining pairs using US Food and Drug Administration (FDA) Adverse Event Reporting System (FAERS) and single centre’s electronic health records (EHR) data. Analyzing data from 2279 RA and 1940 AS patients, we identified 35 significant drug-ADR pairs in RA and 26 in AS, previously unreported in drug labels. Among the novel drug-ADR pairs from KOBIO, 15 were also significant in the FAERS data. Additionally, 2 significant drug-laboratory abnormality pairs were found in RA using CDM MetaLAB analysis. Our findings contribute to the identification of 14 novel drug-ADR signals, expanding our understanding of potential adverse effects related to biological DMARDs and targeted therapies in RA and AS. These results emphasize the importance of ongoing pharmacovigilance for patient safety and optimal therapeutic interventions.

## Introduction

Biological disease-modifying antirheumatic drugs (DMARDs) and targeted therapies are relatively new drugs in the history of rheumatology, and have been used to treat various rheumatic diseases such as rheumatoid arthritis (RA) and ankylosing spondylitis (AS), which exhibit therapeutic effects by antagonizing the actions of various molecules or immune cells^1^. However, they can lead to unexpected adverse effects due to the intricate and systemic nature of the immune system. Therefore, pharmacovigilance for unknown adverse drug reactions (ADRs) is essential^2^. In contrast to drug ADR data from clinical trials conducted in highly controlled environments, drug ADRs have been more comprehensively identified from real-world data, encompassing a wide array of adverse events occurring in diverse patient populations and scenarios, making its significance natably high. Data sources from real-world consist of post-marketing surveillance (PMS), spontaneous reporting systems (SRS), electronic health records (EHRs), claims data, and drug registries where a larger number of patients covered various ages, comorbidities, and concomitant medications.

Pharmaceuticals, physicians, or patients report possible ADRs to the SRS, and several representative SRSs include the World Health Organization's ‘International Pharmacovigilance Program,’ ‘FAERS’, ‘Spontaneous Adverse Drug Reaction Reporting System,’ and ‘Prescription Event Monitoring (PEM)’. A signal, as expressed as “drug-ADR pairs”, indicates the statistical possibility of association between an adverse effect and a suspected drug when the causal relationship is unknown, or the evidence is not yet sufficient^3^. Government organizations for pharmacovigilance or pharmaceuticals generally detect signals through big data mining using measures of disproportionality, such as the proportional reporting risk (PRR), reporting odds ratio (ROR), and Bayesian confidence propagation neural network (BCPNN)^4–6^. The signals are then validated, and some of them are prioritized and assessed for policy establishment for safe drug use.

In contrast, adverse events were observed in the EHRs with clearer causal relationships during patient management. Common data models (CDMs) are well-known standardized EHR data structures and CDMs such as Sentinel CDM or Observation Medical Outcomes Partnership (OMOP)-CDM were originally developed for the pharmacovigilance of newly marketed drugs^7,8^. To date, studies on drug safety using EHR or CDM data have been actively conducted^9–11^. Some authors in this study developed an OMOP-CDM-based pharmacovigilance data-processing pipeline for the active surveillance of laboratory ADR signals and extracted six significant ADR signals causing ear disorders using CDM data^12^.

Other newer resources of the signals could be registries for drugs or diseases run by countries or research networks^13^ where participating researchers at medical institutes register patients and treament-related items with higher accuracy and quality than SRS. In registries, cohorts are established and tracked enabling the inference of the temporal causation of an agent and an ADR.

In summary, registry data, similar to EHR data, holds significance as real-world data and benefits from healthcare professionals' direct reporting of ADRs. Many countries have actively established and collected data from these sources. Given the common limitation of underreporting in all data sources, the exploration of registry data, which has not been widely utilized to identify signals, holds significant value. Thus, this study aimed to discover signals using a registry data from Korea. The KOrean College of Rheumatology BIOlogics & Targeted Therapy Registry (KOBIO) is a nationwide web-based drug registry under the Korean College of Rheumatology (KCR) founded in 2012^14^, where for RA and AS, respectively, they registered 2624 and 2240 patients with 8777 and 8198 followed-ups by October 2021. Signals had not extracted from registry data in rheumatology to our knowledge. In this study, we searched for novel signals of biological DMARDs or targeted therapies in patients with RA or AS using KOBIO data.

## Methods

### Definition of novel signals

**Figure 1:**
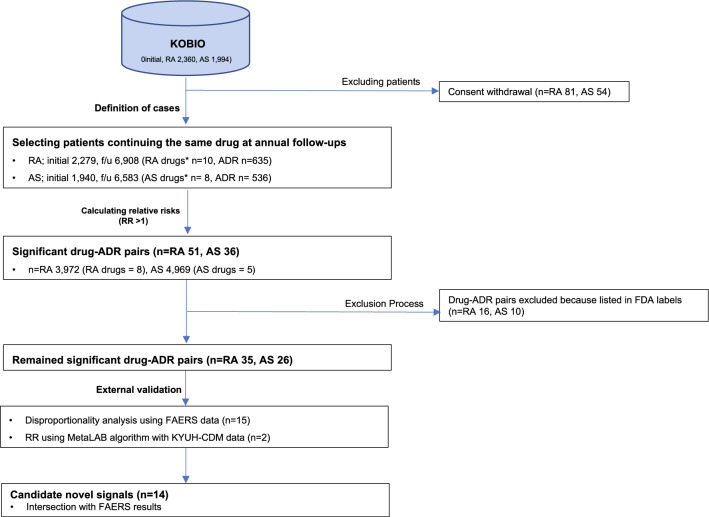
Data collection and overall analysis flow. KOBIO, KOrean College of Rheumatology BIOlogics & Targeted Therapy registry; RA, rheumatoid arthritis; AS, ankylosing spondylitis; ADR, adverse drug reaction; Drug*, drugs by brand name; RR, relative risk; CTZ, certolizumab; BAR, baricitinib; IXK, ixekizumab; RR, relative risk; FDA, Food and Drug Administration; FAERS, Food and Drug Administration Adverse Event Reporting System; CDM, common data model.

The WHO definition of a signal is ‘reported information on a possible causal relationship between and adverse event and a drug. In this study, ‘novel signals’ were defined as statistically significant ADRs of target drugs determined from the source data and were not reported in the latest versions of the FDA labels, either from clinical trials or PMS. We used KOBIO data as the source data and FAERS and KYUH-CDM laboratory data for the external validation of the novel findings. The data collection and overall analysis flow are shown in Fig. 1.

### KOBIO Data collection and preparation for analysis

We retrieved data of patients with RA or AS from the KOBIO database from December 2012 to June 2020. The KOBIO enrolls patient with RA or AS separately when they starts biological DMARDs or targeted therapies or have a switch from one to another, and then collects follow-up data if the patients are on the same agents on yearly base, or when any switching or discontinuation occurs. When patients provide their informed consent, healthcare professionals typically conduct face-to-face interviews with patients to collect comprehensive medical information, including relevant details regarding medications and ADRs. They also review medical charts and laboratory test results. The dataset is primarily segregated by disease (RA or AS) and by phase (initial or follow-ups), incorporating patients' demographic information, risk factors, comorbidities, relevant medication details, laboratory data, and reports of ADRs that occurred during interim periods for the follow-up phase. In KOBIO, they gather data using the brand names of prescribed biologics or targeted therapies, we changed originators and biosimilars into generic names for analysis (see Supplementary Table 1, which demonstrates the drugs and disease codes).

Within the KOBIO registry, adverse drug reactions (ADRs) are systematically categorized according to organ systems, providing the option to choose predefined ADR categories or input additional details in a free-text format. The reporting system obliges the submission of the association between ADRs and the drugs (classified as related, unrelated, or indeterminate), ADR severity (categorized as mild/Grade I, moderate/Grade II, or severe/Grade III), and ADR outcome (indicating whether the ADR did not resolve, resolved, resolved with sequelae, had an unknown outcome, or resulted in death). In light of our study's primary focus on the identification of ADR signals, we specifically selected ADR associations classified as 'related' and 'indeterminate' while excluding ADR severity and outcome data, with our main emphasis placed on the presence or absence of ADR reports.

Furthermore, the KOBIO registry, although primarily serving as a drug registry, separately catalogues data regarding adverse reactions based on the specific diseases. During the data collection phase for our study, medications used in RA and AS, except for TNF inhibitors, did not overlap. The structural differences in the database of the two diseaes and the potential variations in signal based on the specific diseases make it challenging to merge and analyze the data collectively. So, we conducted separate analyses for the RA and AS databases and after obtaining significant drug-ADR paris for both diseases, we merged them and submitted them as candidate signals following the extraction of overlaps with FAERS.

Patients who withdrew consent after registration were excluded from the analysis and each follow-up event was considered an independent case. Only patients who continued treatment with the same agents at follow-up were selected for valid analysis of cause-and-effect relationships. To facilitate external validation of the results with disparate data sources, we converted the ADR terms reported by KOBIO into the Medical Dictionary for Regulatory Activities Preferred Terms (MedDRA PTs).

Total ADRs consisted of ADRs reported by researchers from participating hospitals and laboratory abnormalities collected during follow-up. Contingency tables were used to calculate the relative risk (RR) of each reported ADRs. For this analysis, we designated the number of cases with the drug of interest that also had the ADR of interest as ‘a’, and the number of cases with the drug of interest but without the ADR of interest as ‘b’. Additionally, we labeled the number of ADR cases that occurred with other drugs than interest as ‘c’, and the number of no ADR cases associated with other drugs as ‘d’, categorising these counts by disease. RRs > 1 was considered statistically significant^15^.

$$RR = \frac{{a/\left( {a + b} \right)}}{{c/\left( {c + d} \right)}}$$

**Table:** 

	ADR of interest	No ADR of interest
Drug of interest	a	b
Other drugs	c	d

### Exclusion process for selection of the novel findings

Drug-ADR pairs showing significant RRs (> 1) from the KOBIO data were searched for existence checks in the latest FDA labels of each originator drug (2019–2021). Pairs not included in the reference were selected as signal candidates for further analysis.

### External validation

We conducted external validation of our significant drug-ADR pairs using two different datasets to ensure the reliability and generalizability of the study results and to enhance the overall quality of the research.

#### FAERS database

To externally validate the drug-ADR signals from KOBIO, we used publicly available FAERS data (January 2012 to December 2018, http://www.fda.gov/). In the FAERS database, drug-ADR pairs have been reported with either brand or generic names. We extracted brand names (DrugBank, https://go.drugbank.com/), then changed them into the corresponding generic names (see Supplementary Table 1). For the novel drug-ADR pairs, we performed disproportionality analysis with FAERS data using the R package “PhViD” according to the authors’ protocol (I. Ahmed & A. Poncet, 2016. PhViD: an R package for PharmacoVigilance signal Detection). We obtained the PRR, ROR, and information component (IC) of BCPNN with FDRs for significant drug-ADR pairs (FDR < 0.05).

$$PRR = \frac{{a/\left( {a + b} \right)}}{{c/\left( {c + d} \right)}}\quad ROR = \frac{a/b}{{c/d}}\quad IC = \log_{2} \frac{{a \times \left( {a + b + c + d} \right)}}{{\left( {a + c} \right)\left( {a + b} \right)}}$$

**Table:** 

	ADR of interest “Cases”	Other ADR “Non-cased”
Drug of interest	A	B
Other drugs	c	d

#### KYUH-CDM laboratory data

One of the authors has developed and documented an algorithm for pharmacovigilance within the CDM referred to as MetaLAB^16^. This was designed to identify abnormal test results in response to drug usage for 102 laboratory signals. These signals encompass 38 instances of values exceeding upper limits, 39 instances of values falling below lower limits, and 25 instances of values falling outside the normal range.

In this study, we applied the CDM-based MetaLAB algorithm to KYUH-CDM data (January 2012 to December 2019) to detect ADR signals by employing laboratory test results as supplemental information for ADR assessment using statistical approaches. The patient selection process involved identifying individuals with one of the specified disease codes and one of the relevant drug codes. All drug codes used during the target period were included. Subsequently, patients with or without the drug code of interest were categorized as 'a' and 'c,' respectively, while the presence or absence of laboratory signals of interest was denoted as 'b' and 'd,' respectively (as illustrated in Methods 2.2). The Korean Standard Classification of Diseases (KCD) codes for diseases and the Anatomical Therapeutic Chemical Classification System (ATC) codes for biological DMARDs are shown in Supplementary Table 1. RRs were calculated, and > 1 was considered significant.

### Final selection of candidate signals

To increase the validity of the signals by signal enhancement, the intersection of the KOBIO and FAERS signals was selected and suggested as the final novel signal candidate.

### Statistical analysis

Numerical data are expressed as mean ± standard deviation (SD) or median and interquartile range (IQR), and as numbers (%) for categorical variables. For the KOBIO data and KYUH-CDM, RRs were calculated from contingency tables of “exposure to a drug of interest” and “ADR of interest,” and > 1 was considered significant. When using the FAERS database, we conducted a case/non-case analysis using the PhViD package to calculate PRR, ROR, BCPNN, and FDR. Data mining and statistical analyses were conducted using OracleSQL (for FAERS), PostgreSQL (for KYUH-CDM), and the R software (version 4.1.1).

### Ethics statement

This study was approved by the Institutional Review Board of Konyang University Hospital (IRB No. KY 2020-07-005-003), and complied with the Declaration of Helsinki. The requirement for written informed consent was waived by the approving authority because the patients registered in KOBIO consented to the use of data for research, and the EHR-based CDM data and FAERS dataset comprised of de-identified secondary data.

## Results

### Patients' demographics for the KOBIO data

The total number of patients at initial registration and follow-up, excluding patients who withdrew consent (RA, n = 81; AS, n = 54), was 2279 and 6908 for RA and 1940 and 6583 for AS, respectively (Fig. 1). Among the eight options for the status of drug use at each follow-up, we only selected patients who continued the same medication during follow-ups (RA, n = 3972; AS, n = 4969) to obtain data on temporally related drug ADRs, excluding the effect of other biological DMARDs. Patient’ demographics at the initial registration are shown in Table 1, with the numbers from follow-up. Male to female ratios were 1:4.8 and 3.2:1, and the mean ages of patients were 55.0 ± 13.0 and 39.3 ± 13.2 years for RA and AS respectively. The most commonly used biological DMARDs for RA and AS were tocilizumab and adalimumab, respectively. Ninety-two percent of patients with RA were prescribed concomitant DMARDs, with methotrexate being the most prescribed; 12% of patients with AS were prescribed DMARDs, with both sulfasalazine and methotrexate being the most prescribed drugs.

**Table 1:** Demographics of patients from the KOBIO data.

Category	RA		AS	
Number of patients	Initial: 2279		Initial: 1940	
	Follow-ups: 3972		Follow-ups: 4969	
Gender (M:F)	390 (17.1%):1889 (82.9%)		1448 (76.7%):452 (23.3%)	
Age (mean ± S.D)	55.0 ± 13.0		39.3 ± 13.2	
Symptom duration (y)	8.9 ± 8.0		8.1 ± 8.2	
Disease duration (y)	7.6 ± 7.3		5.0 ± 6.3.0	
Smoking status				
Never smoker	1893 (83.1%)		974 (50.2%)	
Ex-smoker	208 (9.1%)		405 (20.9%)	
Current smoker	178 (7.8%)		561 (28.9%)	
BMI (kg/m^2^)	22.7 ± 3.5		23.6 ± 3.6	
HLA-B27 positivity	NA		1607 (89.5%)	

Biologic DMARDs	Initial	Follow-ups	Initial	Follow-ups
ETN	339 (14.9%)	583 (14.7%)	311 (16.0%)	650 (13.1%)
IFX	224 (9.8%)	328 (8.3%)	431 (22.2%)	1030 (20.7%)
ADA	399 (17.5%)	618 (15.6%)	767 (39.5%)	1867 (37.6%)
GLM	175 (7.7%)	342 (8.6%)	406 (20.9%)	1353 (27.2%)
RTX	21 (0.9%)	106 (2.7%)	NA	NA
ABT	308 (13.5%)	539 (13.6%)	NA	NA
TCZ	573 (25.1%)	1174 (29.6%)	NA	NA
TOF	172 (7.5%)	275 (6.9%)	NA	NA
BAR	68 (3.0%)	7 (0.2%)	NA	NA
SCK	NA	NA	25 (1.3%)	68 (1.4%)
Concomitant NSAID use	NA		1632 (84.1%)	
Aceclofenac	NA		365 (22.4%)	
Celecoxib	NA		397 (24.3%)	
Meloxicam	NA		231 (14.2%)	
Naproxen	NA		359 (22.0%)	
Concomitant csDMARDs use	2116 (92.8%)		232 (12.0%)	
MTX	1847 (87.3%)		121 (52.2%)	
LEF	259 (12.24%)		0 (0%)	
SSZ	163 (7.70%)		133 (57.3%)	
HCQ	226 (10.68%)		2 (0.86%)	
TAC	144 (6.81%)		0 (0%)	
Comorbidities at initial registration				
HTN	670 (29.4%)		307 (15.8%)	
IHD	52 (2.3%)		28 (1.4%)	
Dyslipidemia	471 (20.7%)		260 (13.4%)	
CHF	25 (1.1%)		3 (0.2%)	
Cardiac arrhythmia	25 (1.1%)		16 (0.8%)	
Stroke	20 (0.9%)		6 (0.3%)	
ILD	124 (5.4%)		4 (0.2%)	
COPD	31 (1.4%)		10 (0.5%)	
BA	30 (1.3%)		16 (0.8%)	
Osteoporosis	599 (26.3%)		87 (4.5%)	
DM	280 (12.3%)		95 (4.9%)	
Hyperthyroidism	29 (1.3%)		8 (0.4%)	
Hypothyroidism	103 (4.5%)		16 (0.8%)	
Renal failure	29 (1.3%)		17 (0.9%)	
Electrolyte abnormalities	0 (0%)		1 (0.1%)	
Peptic ulcer	67 (2.9%)		35 (1.8%)	
Liver disease	29 (1.3%)		35 (1.8%)	
Tuberculosis	5 (0.2%)		5 (0.3%)	
Hepatitis B	66 (2.9%)		29 (1.5%)	
Hepatitis C	16 (0.7%)		4 (0.2%)	
HIV	0 (0%)		0 (0%)	
Depression	51 (2.2%)		23 (1.2%)	
Psychosis	5 (0.2%)		5 (0.3%)	
Anemia	972 (42.7%)		419 (21.6%)	
Hematologic malignancy	0 (0%)		0 (0%)	
Solid tumor	16 (0.7%)		21 (1.1%)	
Metastatic cancer	0 (0%)		3 (0.2%)	

KOBIO, KOrean College of Rheumatology BIOlogics & Targeted Therapy registry; RA, rheumatoid arthritis; AS, ankylosing spondylitis; BMI, body mass index; HLA, human leukocyte antigen; NA, not available; NSAID, non-steroidal anti-inflammatory drug; DMARD, disease-modifying anti-rheumatic drug; csDMARDs, conventional synthetic DMARDs; ETN, etanercept; IFX, infliximab; ADA, adalimumab; GLM, golimumab; RTX, rituximab; ABT, abatacept; TCZ, tocilizumab; TOF, tofacitinib; BAR, baricitinib; SCK, secukinumab; MTX, methotrexate; LEF, leflunomide; SSZ, sulfasalazine; HCQ, hydroxychloroquine; TAC, tacrolimus; HTN, hypertension; IHD, ischemic heart disease; CHF, congestive heart failure; ILD, interstitial lung disease; COPD, chronic obstructive pulmonary disorder; BA, bronchial asthma; DM, diabetes mellitus; HIV, human immunodeficiency virus.

### Significant relative risks of drug-ADR pairs from the KOBIO data

We calculated the RRs for each drug (RA, n = 10, AS, n = 8) and ADR (approximately 600), and the signal candidates were presented as “drug-ADR” pairs. For RA, the number of significant drug-ADRs with RR > 1 was 51 from eight agents and 633 ADRs, including 34 laboratory abnormalities (certolizumab not prescribed, and baricitinib unassociated with any significant ADR). For AS, the number of significant ADRs was 36 from four agents and 549 ADRs, including 33 laboratory abnormalities (certolizumab, ixekizumab not prescribed).

### Exclusion process for selection of the novel findings

All candidate ADRs were searched in the latest FDA labels of the originators for the exclusion process. Sixteen drug-ADR pairs for RA and 10 for AS were reported in the labels from the observations in the clinical trials and/or PMS (see Supplementary Table 2, which demonstrates excluded KOBIO drug-ADR pairs previously reported in the latest FDA labels). The final 35 drug-ADR pairs with significant relative risks and 95% confidence interval (CI) for RA and 26 for AS are listed in Tables 2 and 3, respectively.

**Table 2:** Drug-ADR pairs with the significant RRs for RA from the KOBIO data.

Drug	ADRs (MedDRA PT)	KOBIO				FAERS							KYUH-CDM
		Count	RR	95% CI		BCPNN			PRR	FDR	ROR	FDR	
						Count	Expected count	FDR					
ETN	Vitamin D deficiency	4	1.007	1.000	1.014	365	411.9	0.303	0.859	0.448	0.859	0.448	
	Uterine leiomyoma	8	1.013	1.003	1.023	184	329.9	0.493	0.497	0.584	0.496	0.584	
	Renal mass	4	1.007	1.000	1.014	29	58.5	0.397	0.436	0.513	0.435	0.513	
IFX	Blood creatinine increased$	30	1.040	1.004	1.079	209	391.4	0.546	0.505	0.592	0.502	0.592	
	Blood creatinine decreased$	28	1.052	1.016	1.089	25	31.0	0.161	0.787	0.308	0.787	0.308	
ADA	Dry eye	7	1.009	1.000	1.017	1557	1892.3	0.482	0.752	0.591	0.749	0.590	
	White matter lesion	4	1.007	1.000	1.013	118	60.5	0.000*	3.917	0.000*	3.921	0.000*	
	Albuminuria$	28	1.091	1.006	1.182	2	0.7	0.023*	Inf	0.645	Inf	0.645	
GLM	Iron deficiency anemia	5	1.013	1.000	1.027	81	44.2	0.000*	1.870	0.000*	1.879	0.000*	
	Diabetic retinopathy	5	1.015	1.002	1.028	NA	NA	NA	NA	NA	NA	NA	
	Vitreous floaters	4	1.012	1.000	1.024	NA	NA	NA	NA	NA	NA	NA	
	Gastroesophageal reflux disease	8	1.017	1.000	1.034	156	185.4	0.306	0.838	0.416	0.835	0.416	
	Spinal compression fracture	6	1.016	1.002	1.031	63	32.1	0.000*	2.011	0.000*	2.020	0.000*	
	Fibromyalgia	7	1.019	1.003	1.034	321	237.3	0.000*	1.365	0.000*	1.381	0.000*	
	Sjogren's syndrome	8	1.017	1.001	1.035	81	49.3	0.000*	1.679	0.000*	1.686	0.000*	
	Endometriosis	5	1.014	1.001	1.027	25	18.6	0.008*	1.355	0.024*	1.356	0.024*	
	Tremor	6	1.018	1.003	1.032	226	221.3	0.035*	1.022	0.132	1.022	0.132	
	High density lipoprotein cholesterol increased$	62	1.250	1.000	1.563	NA	NA	NA	NA	NA	NA	NA	
ABT	Anemia	68	1.041	1.006	1.077	886	936.6	0.228	0.943	0.365	0.940	0.365	
	Cataract	10	1.013 ara>	1.001	1.025	1172	746.1	0.000*	1.623	0.000*	1.670	0.000*	
	Gastroesophageal reflux disease	8	1.014	1.004	1.025	331	403.5	0.388	0.812	0.493	0.808	0.493	
	Hypercholesterolemia	6	1.010	1.001	1.020	64	80.3	0.256	0.788	0.384	0.787	0.384	
	Depression	8	1.011	1.000	1.022	984	1030.8	0.189	0.952	0.331	0.949	0.331	
	Asthma	9	1.014	1.002	1.025	515	421.7	0.000*	1.237	0.000*	1.244	0.000*	
	Interstitial lung disease	13	1.014	1.000	1.028	971	379.2	0.000*	2.806	0.000*	2.918	0.000*	
	Anemia$	133	1.068	1.014	1.124	886	936.6	0.228	0.943	0.365	0.940	0.365	
	Blood glucose increased$	137	1.092	1.033	1.156	243	300.7	0.377	0.799	0.478	0.796	0.478	
TCZ	Leukocytosis	4	1.003	1.000	1.007	68	74.0	0.104	0.909	0.240	0.909	0.240	
	Duodenal ulcer	4	1.003	1.000	1.007	30	40.4	0.235	0.719	0.362	0.719	0.362	
	Osteoporosis	97	1.030	1.010	1.050	2275	1132.4	0.000*	2.291	0.000*	2.384	0.000*	
	Adenomyosis	7	1.006	1.001	1.010	7	12.1	0.205	0.552	0.327	0.552	0.327	
	Alopecia	18	1.012	1.005	1.020	2790	3346.0	0.561	0.817	0.598	0.801	0.598	
	Leukopenia$	143	1.087	1.062	1.112	NA	NA	NA	NA	NA	NA	NA	X
	Thrombocytopenia$	36	1.019	1.008	1.030	NA	NA	NA	NA	NA	NA	NA	
TOF	Thrombocytosis$	16	1.033	1.002	1.064	52	21.6	0.000*	2.616	0.000*	2.621	0.000*	X

Laboratory abnormalities were marked with $ and FAERS ADR with FDR < 0.05 were marked with *

KOBIO, KOrean College of Rheumatology BIOlogics & Targeted Therapy registry; RA, rheumatoid arthritis; AS, ankylosing spondylitis; ETN, etanercept; IFX, infliximab; ADA, adalimumab; GLM, golimumab; RTX, rituximab; ABT, abatacept; TCZ, tocilizumab; TOF, tofacitinib; MedDRA PT, medical dictionary for regulatory activities preferred term; RR, relative risk; C.I. , confidence interval; BCPNN, Bayesian confidence propagation neural network; PRR, proportional reporting ratio; ROR, reporting odds ratio; FDR, false discovery rate; CDM, common data model; FDA, food and drug administration; NA, not available.

**Table 3:** Drug-ADR pairs with the significant RRs for AS from the KOBIO data.

Drug	ADRs (MedDRA PT)	KOBIO				FAERS							KYUH-CDM
		Count	RR	95% CI		BCPNN			PRR	FDR	ROR	FDR	
						Count	Expected count	FDR					
ETN	Gingival ulceration	9	1.011	1.002	1.021	17	14.0	0.034*	1.334	0.082	1.334	0.082	
	Depression	11	1.012	1.001	1.022	3920	4398.3	0.496	0.851	0.606	0.842	0.606	
	Blood glucose increased$	14	1.017	1.004	1.030	1978	1532.3	0.469	0.785	0.582	0.782	0.582	
IFX	Iron deficiency anemia	8	1.006	1.001	1.012	186	193.6	0.131	0.954	0.296	0.954	0.296	
	Periodontal disease	7	1.007	1.001	1.012	18	20.4	0.137	0.865	0.289	0.864	0.289	
	Hepatic cirrhosis	4	1.004	1.000	1.008	308	367.2	0.378	0.816	0.495	0.814	0.495	
	Blood in urine$	93	1.074	1.029	1.121	NA	NA	NA	NA	NA	NA	NA	
ADA	Presyncope	4	1.002	1.000	1.004	436	463.6	0.184	0.900	0.369	0.900	0.369	
	Benign prostatic hyperplasia	9	1.004	1.001	1.007	282	191.3	0.000*	2.281	0.000*	2.284	0.000*	
	Blood creatinine increased$	292	1.032	1.007	1.057	865	869.6	0.091	0.991	0.213	0.991	0.213	
GLM	Conjunctivitis	7	1.004	1.000	1.008	73	57.5	0.002*	1.282	0.010*	1.284	0.010*	
	Abdominal pain	6	1.004	1.001	1.008	554	1029.6	0.560	0.529	0.632	0.496	0.634	
	Enthesopathy	18	1.009	1.002	1.015	NA	NA	NA	NA	NA	NA	NA	
	Rotator cuff syndrome	4	1.003	1.000	1.006	152	143.3	0.028*	1.063	0.104	1.064	0.104	
	Nephropathy	4	1.003	1.000	1.006	1	12.3	0.356	0.079	0.450	0.079	0.450	
	Proteinuria	5	1.004	1.000	1.007	18	15.9	0.042*	1.136	0.133	1.136	0.133	
	Rhinitis allergic	10	1.005	1.001	1.010	38	11.1	0.000*	3.738	0.000*	3.750	0.000*	
	Dermatitis	18	1.008	1.002	1.015	15	55.5	0.466	0.263	0.551	0.262	0.551	
	Skin depigmentation	4	1.003	1.000	1.006	2	6.3	0.257	0.309	0.363	0.309	0.363	
	White blood cell count increased$	121	1.025	1.006	1.044	73	118.5	0.433	0.608	0.522	0.604	0.522	
	Hematocrit increased$	52	1.015	1.003	1.027	NA	NA	NA	NA	NA	NA	NA	
	Blood creatinine decreased$	27	1.009	1.001	1.018	1	6.4	0.296	0.151	0.405	0.151	0.405	
	Blood cholesterol increased$	437	1.101	1.045	1.161	107	150.6	0.398	0.703	0.509	0.699	0.509	
	Low density lipoprotein cholesterol increased$	199	1.096	1.034	1.162	3	8.5	0.274	0.344	0.395	0.344	0.395	
	Blood triglycerides increased$	269	1.088	1.009	1.173	26	27.7	0.108	0.937	0.229	0.937	0.229	

Laboratory abnormalities were marked with $ and FAERS ADR with FDR < 0.05 were marked with *

KOBIO, KOrean College of Rheumatology BIOlogics & Targeted Therapy registry; RA, rheumatoid arthritis; AS, ankylosing spondylitis; ETN, etanercept; IFX, infliximab; ADA, adalimumab; GLM, golimumab; MedDRA PT, medical dictionary for regulatory activities preferred term; RR, relative risk; C.I., confidence interval; BCPNN, Bayesian confidence propagation neural network; PRR, proportional reporting ratio; ROR, reporting odds ratio; FDR, false discovery rate; CDM, common data model; FDA, food and drug administration; NA, not available.

### External validation

#### FAERS database

For external validation, the final drug-ADR pairs from 3.3 were searched in the FAERS database with generic names, and disproportionality analyses were conducted. All PRR and ROR results were identical, and the BCPNN results included all PRR and ROR results for both the RA and AS (Tables 2 and 3). For RA, 13 of 35 drug-ADR pairs were also significant in the FAERS dataset (any of BCPNN, PRR, and ROR), and for AS, 6 of 26 pairs were common and are presented as marked in the tables.

#### KYUH-CDM data

Using the codes for diseases and drugs in Supplementary Table 1, we retrieved 1411 patients with RA and 656 patients with AS from the CDM data. When we independently applied the MetaLAB algorithm to the patients, the significant drug–laboratory ADR pairs (RR > 1, *p* < 0.05) were 14 pairs for RA, and 9 pairs for AS. The results consistent with the KOBIO results were “TCZ-Anemia” and “TOF-Thrombocytosis,” observed for RA (see Supplementary Table 2).

### The final selection of candidate signals

The intersection of the significant signals from KOBIO and FAERS (BCPNN, PRR, and ROR) was selected as the final candidate (Tables 2 and 3), and the final 14 drug-ADR pairs are listed in Table 4.

**Table 4:** Final drug-ADR signals suggested from the KOBIO data.

Drugs	Signals
ADA (2)	White matter lesion, Benign prostatic hyperplasia
GLM (7)	Iron deficiency anemia, Spinal compression fracture, Fibromyalgia, Sjogren's syndrome, Endometriosis, Conjunctivitis, Rhinitis allergic
ABT (3)	Cataract, Asthma, Interstitial lung disease
TCZ (1)	Osteoporosis
TOF (1)	Thrombocytosis

ADR, adverse drug reaction; KOBIO, KOrean College of Rheumatology BIOlogics & Targeted Therapy registry; ADA, adalimumab; GLM, golimumab; ABT, abatacept; TCZ, tocilizumab; TOF, tofacitinib.

## Discussion

ADR is a serious problem that incurs various costs and losses to both patients and society, and drugs should be properly monitored for ADRs. For decades, quantitative measurements, such as the signal detection of ADRs, have been conducted, particularly for newer drugs^17,18^. Several drugs have been withdrawn from the market through signal detection with SRSs^19,20^. The biological DMARDs manufactured from biological sources using recombinant techniques have been widely prescribed for treating various rheumatic diseases, they also mandate comprehensive and thorough pharmacovigilance^3^. The main data source of pharmacovigilance is SRS, and other longitudinal real-world data sources are EHRs, and claims data. Additionally, multimodal signal detection was performed to obtain more valid results by combining the results from the SRSs, claims data, and EHRs^21–24^. Another data resource for ADRs are registries, and examples of nationwide registries for biological drugs are BSRBR(British Society for Rheumatology Biologics Register), DANBIO(Danish Database for Biological Therapies in Rheumatology), and ARTIS (antirheumatic therapies register, the Swedish biologics register). They include many patients on biological DMARDs nationwide, and could be valuable resources for signal detection. However, research on signal extraction from drug registry data has not yet been actively conducted.

This is the first report of signal detection for biological DMARDs from a nationwide drug registry, with external validation using two different datasets: FAERS and single-centre CDM laboratory data. Before detecting the signals of the drugs from the KOBIO data, we first assessed the performance of extracting known drug-ADRs. We found that several previously reported ADRs of several drugs were extracted from KOBIO; for example, acute nasopharyngitis was extracted for etanercept and golimumab, anemia for etanercept, golimumab, and tofacitinib, and hyperlipidemia for tocilizumab (see Supplementary Table 2).

The identification of the final 14 drug-ADRs as signals was mainly performed by calculation using operational definitions with statistical significance. Therefore, there is no related literature by definition, and we searched for mutations with traits similar to the relevant signal in the genes affected by each drug to seek possible associations. Tofacitinib-thrombocytosis was the only drug-ADR pair that was significant in all KOBIO, FAERS, and CDM results, and tofacitinib is a well-known drug that targets JAK 1,2,3 and TYK2. Although tofacitinib mainly inhibits JAK3 and JAK1 as its main targets, it also inhibits JAK2 to a lesser extent; therefore, it decreases hematopoiesis via thrombopoietin, erythropoietin, and GM-CSF^25^. However, thrombocytosis was observed in this study and when we searched in the GWAS catalog (https://www.ebi.ac.uk/gwas/), rs150221602-C, rs149757596-C, rs150221602-C, rs41215003-A, rs41316003-A, rs150221602-C, rs149757596-C, rs150221602-C, rs776830350 -?, rs150221602-?, rs77375493-T, rs41316003-A, rs41316003-A, and rs41316003-? were associated with JAK2, and mutations associated with thrombocytosis were identified, leaving room for potential drug relationships. As a subsequent process, it is crucial to determine the priorities of signals for evaluation, and they should undergo further external validation through reviews of SRSs, EHRs, literature, experimental trials, and the study of relevant mechanisms^17,26–28^.

This study shares common limitations inherent to pharmacovigilance studies, including constraints stemming from insufficient data sources, underreporting, small sample sizes, and varying report quality. In addition to these general limitations, several study-specific limitations should be noted. First, an initial constraint was our inability to integrate the datasets for TNF inhibitors in both diseases from the outset. Instead, the merging occurred at a later stage when combining them with the FAERS signal. Second, our study included only cases where the same medication was continued until the next follow-up, excluding instances of medication discontinuation or switching to another drug registered in KOBIO. This decision was based on the common practice of switching biological DMARDs or targeted therapies due to inefficacy without wash-out periods, which can complicate accurate ADR assessments. Third, for external validation, we utilized laboratory data from a single center's CDM with the MetaLAB, as the conversion of EHR data into CDM was in progress during the data acqusition period. To improve the study's overall validity and quality, future analyses should incorporate CDM data including majority of EHR data from multiple hospitals across South Korea. Furthermore, it's worth noting that for analysis purposes, we categorized drugs based on their generic names. However, it's important to acknowledge that biosimilars may not necessarily share the same adverse drug reactions (ADRs) as their originator drugs^3,29^.

In conclusion, we identified 14 novel drug-ADR signals of biological DMARDs and targeted therapies in the rheumatology field from KOBIO data. Further evaluation and external validation using other databases and literature should be conducted to assess the conclusive causal relationships between these drug-ADR signals.

### Supplementary Information

Supplementary Table 1.

Supplementary Table 2.

## Data Availability

The data included in this article cannot be shared publicly because KOBIO data are provided by the Korean College of Rheumatology(KCR) to KCR members through research proposal contests and reviews. The data will be shared upon reasonable request by the corresponding author.

## References

[1] Lin YJ, Anzaghe M, Schülke S (2020). Update on the pathomechanism, diagnosis, and treatment options for rheumatoid arthritis. Cells.

[2] Ingrasciotta Y (2018). Safety of biologics, including biosimilars: Perspectives on current status and future direction. Drug Saf..

[3] Safety of Medicines: A Guide to Detecting and Reporting Adverse Drug Reactions. (WHO, 2002). http://whqlibdoc.who.int/hq/2002/WHO_EDM_QSM_2002.2.pdf

[4] Meyboom RHB (1997). Principles of signal detection in pharmacovigilance. Drug Saf..

[5] Evans SJ, Waller PC, Davis S (2001). Use of proportional reporting ratios (PRRs) for signal generation from spontaneous adverse drug reaction reports. Pharmacoepidemiol. Drug Saf..

[6] Bate A (1998). A Bayesian neural network method for adverse drug reaction signal generation. Eur. J. Clin. Pharmacol..

[7] Platt R (2012). The U.S. Food and Drug Administration's Mini-Sentinel program: Status and direction. Pharmacoepidemiol. Drug Saf..

[8] Stang PE (2010). Advancing the science for active surveillance: rationale and design for the observational medical outcomes partnership. Ann. Intern. Med..

[9] Dimitriadis VK, Gavriilidis GI, Natsiavas P (2021). Pharmacovigilance and Clinical Environment: Utilizing OMOP-CDM and OHDSI Software Stack to Integrate EHR Data.

[10] Yu Y (2019). ADEpedia-on-OHDSI: A next generation pharmacovigilance signal detection platform using the OHDSI common data model. J. Biomed. Inform..

[11] Rho MJ (2016). Common data model for decision support system of adverse drug reaction to extract knowledge from multi-centre database. Inf. Technol. Manag..

[12] Shin H (2021). An OMOP-CDM based pharmacovigilance data-processing pipeline (PDP) providing active surveillance for ADR signal detection from real-world data sources. BMC Med. Inform. Decis. Mak..

[13] Mcneil JJ, Piccenna L, Ronaldson K, Ioannide-Demos LL (2010). The value of patient-centred registries in phase IV drug surveillance. Pharmaceut. Med..

[14] Kim J (2021). KOBIO, the first web-based korean biologics registry operated with a unified platform among distinct disease entities. J. Rheum Dis..

[15] Faillie JL (2019). Case-non-case studies: Principle, methods, bias and interpretation. Therapie.

[16] Lee S (2017). Standard-based comprehensive detection of adverse drug reaction signals from nursing statements and laboratory results in electronic health records. J. Am. Med. Inform. Assoc..

[17] Bate A, Hornbuckle K, Juhaeri J, Motsko SP, Reynolds RF (2019). Hypothesis-free signal detection in healthcare databases: finding its value for pharmacovigilance. Ther. Adv. Drug Saf..

[18] Hauben M, Zhou X (2003). Quantitative methods in pharmacovigilance. Drug Saf..

[19] Olivier P, Montastruc JL (2006). The nature of the scientific evidence leading to drug withdrawals for pharmacovigilance reasons in France. Pharmacoepidemiol. Drug Saf..

[20] Clarke A, Deeks JJ, Shakir SAW (2006). An assessment of the publicly disseminated evidence of safety used in decisions to withdraw medicinal products from the UK and US markets. Drug Saf..

[21] Harpaz R (2013). Combing signals from spontaneous reports and electronic health records for detection of adverse drug reactions. J. Am. Med. Inform. Assoc..

[22] Harpaz R (2017). Toward multimodal signal detection of adverse drug reactions. J. Biomed. Inform..

[23] Li Y, Ryan PB, Wei Y, Friedman C (2015). A method to combine signals from spontaneous reporting systems and observational healthcare data to detect adverse drug reactions. Drug Saf..

[24] Lee S, Cha J, Kim JY, Son GM, Kim DK (2021). Detection of unknown ototoxic adverse drug reactions: An electronic healthcare record-based longitudinal nationwide cohort analysis. Sci. Rep..

[25] Yoshida K, Solomon DH, Kim SY (2015). Active-comparator design and new user design in observational studies. Nat. Rev. Rheumatol..

[26] Bissonnette R (2021). Signal detection and methodological limitations in a real-world registry: Learnings from the evaluation of long-term safety analyses in PSOLAR. Drug Saf..

[27] Malikova MA (2020). Practical applications of regulatory requirements for signal detection and communications in pharmacovigilance. Ther. Adv. Drug Saf..

[28] Caster O, Aoki Y, Gattepaille LM, Grundmark B (2020). Disproportionality analysis for pharmacovigilance signal detection in small databases or subsets: Recommendations for limiting false-positive associations. Drug Saf..

[29] Trifirò G, Marcianò I, Ingrasciotta Y (2018). Interchangeability of biosimilar and biological reference product: Updated regulatory positions and pre- and post-marketing evidence. Expert Opin. Biol. Ther..

