# Supplementary material for: Detection of novel drug-adverse drug reaction signals in rheumatoid arthritis and ankylosing spondylitis: analysis of Korean real-world biologics registry data
Source: Sci Rep. 2024 Feb 1;14:2660. doi: 10.1038/s41598-024-52822-w (PMC10834537; doi:10.1038/s41598-024-52822-w)
Supplement: Supplementary file 1 — Supplementary Table 1. [file 41598_2024_52822_MOESM1_ESM.pdf]

**Supplementary Table 1. Drugs and disease names or codes used for retrieval and analysis**

| Data | Category | Names / Codes |
|---|---|---|
| **KOBIO** | RA drugs | Enbrel®, Etoloce® (previous Brenzys®), Eucept® → ETN; Remicade®, Remsima®, Remaloce® (previous Renflexiss®) → IFX; Humira® →ADA; Simponi® → GLM; Cimzia® → CTZ; Mabthera®, Truxima ® → RTX; Orencia® → ABT; Actemra® →TCZ; Xeljanz® → TOF; Olumiant ® → BAR |
| | AS drugs | Enbrel®, Etoloce® (previously Brenzys®), Eucept® → ETN; Remicade®, Remsima®, Remaloce® (previous Renflexiss®) → IFX; Humira® → ADA; Simponi® → GLM; Cimzia® → CTZ; Cosentyx® → SCK, Taltz® → IXK |
| **FAERS Database** | Drugs | Etanercept, Enbrel ®, Eticovo® → ETN; Infliximab, Avsola®, Flixabi®, Inflectra®, Remicade®, Renflexis® → IFX; Adalimumab, Humira® →ADA ; Golimumab, Simponi® → GLM; Rituximab, Rituxan®/MabThera®, Rituxan Hycela®, Riabni®, Ruxience®, Truxima® → RTX ; Abatacept, Orencia® → ABT ; Tocilizumab, Actemra®, RoActemra® → TCZ; Tofacitinib, Xeljanz® → TOF; Secukinumab, Cosentyx® → SCK |
| **KYUH-CDM data** | KCD codes | RA; M050, M053, M058, M059<br>AS; M450, M451, M452, M453, M454, M455, M456, M457, M458, M459 |
| | Drug ATC codes | ETN (L04AB01)→ PXETC5P, PXETC5, PXETC5Y, PXETC, PXETC1; IFX (L04AB02)→ PXIFX, PXIFXM; ADA (L04AB04)→ PXADLM-1, PXADLM; GLM (L04AB06)→ PXGMMIV, PXGMM; TCZ (L04AC07)→ PXTCM20, PXTCM40, PXTCM8; ABT (L04AA24)→ PXABTSC, PXABT; RTX (L01XC02)→ PXRTX, PXRTX5, PXRTXS, PXRTXS5; TOF (L04AA29)→ PHOTFCTN, PTFCTN10; BAR (L04AA37)→ PBRCT2, PBRCT4; SCK (L04AC10)→ PXSKCNSR, PXSKCNM |

KOBIO; KOrean College of Rheumatology BIOlogics & Targeted Therapy registry, FAERS; Food and drug administration Adverse Event Reporting System, CDM; common data model, RA; rheumatoid arthritis, AS; ankylosing spondylitis, ETN; etanercept, IFX; infliximab, ADA; adalimumab, GLM; golimumab, CTZ; certolizumab, RTX; rituximab, ABT; abatacept, TCZ; tocilizumab, TOF; tofacitinib, BAR; baricitinib, SCK; secukinumab, IXK; ixekizumab, KCD; Korean Standard Classification of Disease, ATC; Anatomical Therapeutic Chemical Classification System
