# Supplementary material for: Detection of novel drug-adverse drug reaction signals in rheumatoid arthritis and ankylosing spondylitis: analysis of Korean real-world biologics registry data
Source: Sci Rep. 2024 Feb 1;14:2660. doi: 10.1038/s41598-024-52822-w (PMC10834537; doi:10.1038/s41598-024-52822-w)
Supplement: Supplementary file 2 — Supplementary Table 2. [file 41598_2024_52822_MOESM2_ESM.pdf]

Supplementary Table 2. Excluded KOBIO drug-ADR pairs previously reported in the latest FDA labels

Drug	ADRs (MedDRA PT)	KOBIO			FAERS					Disease
		RR	95% C.I.		BCPNN	PRR	FDR	ROR	FDR	
ETN	Acute nasopharyngitis	1.03	1.01	1.05	NA	NA	NA	NA	NA	RA
	Psoriasis	1.01	1.00	1.02	0.00*	3.04	0.00*	4.23	0.00*	RA
	White blood cell count decreased	1.02	1.00	1.04	0.00*	1.48	0.00*	1.49	0.00*	AS
IFX	Tachycardia	1.01	1.00	1.01	0.00*	4.92	0.00*	5.09	0.00*	AS
	Constipation	1.00	1.00	1.01	0.48	0.82	0.58	0.82	0.58	AS
	Pain in extremity	1.01	1.00	1.01	0.57	0.37	0.66	0.30	0.66	AS
	Hemoglobin decreased	1.02	1.00	1.04	0.00*	1.26	0.00*	1.27	0.00*	AS
ADA	Hematuria	1.09	1.00	1.18	0.43	0.74	0.55	0.74	0.55	RA
	Blood alkaline phosphatase increased	1.03	1.01	1.05	0.10	0.94	0.26	0.94	0.26	RA, AS
	Alopecia	1.00	1.00	1.01	0.53	0.68	0.66	0.66	0.65	AS
GLM	Acute nasopharyngitis	1.04	1.01	1.07	NA	NA	NA	NA	NA	RA
	Anemia	1.09	1.02	1.16	0.00*	1.15	0.00*	1.16	0.00*	RA
	Rash	1.01	1.00	1.02	0.01*	1.04	0.04*	1.04	0.05*	AS
	Hemoglobin increased	1.02	1.01	1.03	0.20	0.63	0.32	0.63	0.32	AS
	Liver function test abnormal	1.04	1.01	1.07	0.46	0.61	0.55	0.60	0.55	AS
RTX	Blood glucose increased	1.31	1.11	1.55	0.00*	2.24	0.00*	2.29	0.00*	RA
TCZ	Hyperlipidemia	1.08	1.04	1.11	0.10	0.92	0.24	0.92	0.24	RA
	Leukopenia	1.09	1.06	1.11	0.00*	2.35	0.00*	2.40	0.00*	RA
	Thrombocytopenia	1.02	1.01	1.03	0.07	0.99	0.20	0.99	0.20	RA
	Alanine aminotransferase increased	1.04	1.01	1.06	0.00*	2.34	0.00*	2.40	0.00*	RA
	Blood cholesterol increased	1.09	1.03	1.15	0.00*	3.75	0.00*	4.06	0.00*	RA
	Blood triglycerides increased	1.08	1.03	1.14	0.00*	2.24	0.00*	2.25	0.00*	RA
	High density lipoprotein cholesterol increased	1.57	1.26	1.97	NA	NA	NA	NA	NA	RA
TOF	Anemia	1.07	1.02	1.12	0.01*	1.06	0.01*	1.06	0.02*	RA
	High density lipoprotein cholesterol increased	1.57	1.26	1.97	NA	NA	NA	NA	NA	RA

Laboratory abnormalities were shaded, and FAERS ADR with FDR <0.05 were shaded*.

KOBIO; KOrean College of Rheumatology BIOlogics & Targeted Therapy registry, RA; rheumatoid arthritis, AS; ankylosing spondylitis, ETN; etanercept, IFX; infliximab, ADA; adalimumab, GLM; golimumab, RTX; rituximab, ABT; abatacept, TCZ; tocilizumab, TOF; tofacitinib, MedDRA PT; medical dictionary for regulatory activities preferred term, RR; relative risk, C.I.; confidence interval, BCPNN; Bayesian confidence propagation neural network, PRR; proportional reporting ratio, ROR; reporting odds ratio, FDR; false discovery rate, CDM; common data model, FDA; food and drug administration, NA; not available
